# Carriage of antimicrobial‐resistant *E. coli* in urban stray cats (*Felis catus* *)*

**DOI:** 10.1111/avj.70118

**Published:** 2026-07-21

**Authors:** JS Kuan, S Mukerji, D Hampson, S Abraham

**Affiliations:** ^1^ School of Veterinary Medicine Murdoch University Perth Australia; ^2^ School of Medical, Molecular & Forensic Sciences Murdoch University Perth Australia

**Keywords:** antimicrobial susceptibility, Australia, *Escherichia coli*, extended‐spectrum β‐lactamase, minimum inhibitory concentration, Multidrug resistance, One Health, urban stray cats

## Abstract

Antimicrobial resistance (AMR) in *Escherichia coli* is a growing concern in both human and veterinary medicine, driven by the inappropriate use of antimicrobials. Companion animals may act as community reservoirs and sentinels for antimicrobial‐resistant bacteria due to their close contact with humans and other animals. However, resistance carriage in urban stray cats is poorly defined, particularly as many have limited direct antimicrobial exposure. This population provides an opportunity to assess baseline antimicrobial resistance carriage in companion animals with relatively low selection pressure. This study aimed to investigate the carriage of antimicrobial‐resistant *E. coli* in the stray cat population in Perth, Western Australia. A total of 72 faecal samples from urban stray cats (May 2024–November 2024) were screened for carriage of antimicrobial‐resistant *E. coli*. Chromogenic selective agars infused with antimicrobials were used to detect resistance to ampicillin (first line) and critically important antimicrobials (CIA), including ciprofloxacin and extended‐spectrum cephalosporins. Presumptive *E. coli* were confirmed by matrix‐assisted laser desorption/ionization time‐of‐flight mass spectrometry (MALDI‐TOF MS) and tested by broth microdilution using the Robotic Antimicrobial Susceptibility Platform (RASP). Screening of 72 faecal samples yielded *E. coli* growth from 69 samples (95.8%). Of these, 37.7% (26/69) carried ampicillin‐resistant *E. coli*, 1.4% (1/69) exhibited an extended‐spectrum beta‐lactamases phenotype, and no ciprofloxacin‐resistant *E. coli* were detected. A total of 27 *E. coli* isolates underwent MIC testing, showing resistance to ampicillin (100%), cefotaxime (7.4%), ceftazidime (7.4%), sulfamethoxazole (33.3%), tetracycline (37.0%), and trimethoprim (29.6%). Ampicillin‐resistant *E. coli* was common among urban stray cats, while no ciprofloxacin resistance was detected, suggesting low immediate CIA‐related risk. However, the presence of extended‐spectrum cephalosporin resistance, albeit at low levels, warrants further investigation given the potential for transmission and dissemination via mobile genetic elements. These findings indicate that urban stray cats can carry and potentially spread resistant bacteria despite their limited opportunities for antimicrobial exposure.

Antimicrobial resistance is a critical issue affecting both humans and animals, where the overuse and misuse of antimicrobials have led to a recent rise in antimicrobial resistance (AMR). The epidemiology of resistance is intricate, with factors beyond the number and dosage of antimicrobials used playing a role.^1^ Strong selective pressures on the human and animal microbiota, as well as the antimicrobial‐filled environment, have significantly changed the evolution and spread of AMR, and this has led to the transfer of many AMR strains among disease‐causing bacterial species.^2^

*Escherichia coli* (*E. coli*) is a ubiquitous bacteria found in the environment and commensally in the gut of healthy animals including cats.^3^ However, it can also cause severe infections and serve as a significant reservoir of resistance genes. *E. coli* is naturally susceptible to nearly all clinically relevant antimicrobial agents, but it has a considerable ability to acquire resistance genes.^4^ This makes it a suitable indicator organism for studying the occurrence and prevalence of AMR.

Antimicrobial‐resistant *E. coli* have been found in hospitalised animals and within veterinary settings, where the occurrence of inhibitor‐resistant and extended‐spectrum beta‐lactamases (ESBL) has been identified.^4^, ^5^, ^6^, ^7^ The usage of broad‐spectrum antibiotics as first‐line treatment has subjected hospitalised pets to high selective pressure for antimicrobial resistance.^6^ There is, however, little research on AMR status in companion animals with low selective pressure, such as the stray population that experience less opportunities for exposure to humans and antimicrobials.

Studying urban stray cats can provide valuable epidemiological data, as they often have diverse backgrounds and exposure histories,^8^ making them representative of the overall environmental prevalence of resistant bacteria. Stray animals often act as reservoir hosts for bacteria and are a bridge between pet and feral populations. With the population of urban stray cats increasing over the years, and with densities much higher in urban areas,^9^ the frequency of interactions between roaming pet cats and strays is further amplified. As urban stray cats interact with wildlife and pet cats that roam outdoors, they may play a part in the spread of antimicrobial‐resistant strains, worsening the current worldwide issue.^10^ These cats may come into close contact with humans as well, which heightens the risk of direct zoonotic transmission of resistant bacteria, posing a threat to public health. However, there have been few studies examining the role of stray companion animals in carrying and spreading resistance to broad‐spectrum antimicrobials used in both veterinary and human medicine.^10^ Hence, understanding the prevalence of antimicrobial‐resistant *E. coli* in urban stray cats can help identify future risks to humans. In addition, two studies from China and Italy respectively found that the faeces of healthy cats were a significant reservoir of ESBL‐producing *E. coli*,^11^, ^12^, ^13^ which then could be transmitted to humans. ESBL enzymes hydrolyze third‐generation cephalosporins and because of this are now considered one of the most severe public health concerns.^14^

Urban stray cats occupy the human–animal–environment interface and may act as sentinels of environmental antimicrobial resistance (AMR). This is particularly relevant for populations presumed to experience low antimicrobial selection pressure, including young stray animals with limited or no documented direct antimicrobial exposure. However, even without direct antimicrobial treatment, such animals may acquire resistant bacteria through indirect exposure to wildlife, urban environments, or other human‐associated sources. Despite this potential importance, very little work has focused on urban stray cat populations, which was the specific focus of this study. Defining the occurrence of resistant *E. coli* in this population may strengthen One Health risk assessment and support efforts to preserve antimicrobial efficacy in both veterinary and human medicine.

The aim of this study was to investigate the carriage of antimicrobial‐resistant *E. coli* in urban stray cats from the Perth region. The objectives were to determine the prevalence of resistant *E. coli and* to characterise recovered isolates phenotypically and genotypically, particularly for resistance to beta‐lactams, extended‐spectrum cephalosporins, and quinolones. We hypothesised that resistant *E. coli* would be detectable in urban stray cats despite their presumed low direct antimicrobial exposure

## Materials and methods

A cross‐sectional study was conducted over a seven‐month period from May 2024 to November 2024, to estimate the prevalence of AMR in *E. coli* among the sampled stray cat population. This project involved the collection of samples from 72 randomly selected urban stray cats admitted to Cat Haven, the largest animal shelter in Western Australia. The sampled animals were considered representative of the shelter‐admitted stray cat population in Perth. The exclusion criteria were cats that were diseased and/or undergoing antimicrobial therapy treatments. Faecal samples from these urban stray cats brought into the Animal Hospital at Murdoch University (TAHMU) were collected and analysed for the presence of antimicrobial resistance in *E. coli*.

Cats less than a year old were recruited for this study as part of the spay and neuter program by Cat Haven and TAHMU undertaken from May to October 2024. A single faecal sample was collected from each cat before sterilisation during the DVM4 and DVM5 students' spey and neuter labs. Faecal samples were collected directly from the rectum to avoid contamination, using a gloved index finger and were placed in a sterile specimen container containing a drop of sterile saline to keep the samples moist. Samples were stored at 4°C until laboratory analysis at the Antimicrobial Resistance and Infectious Diseases (AMRID) Laboratory at Murdoch University, which was conducted within 48 h.

### Sampling considerations

The ethical aspects considered for this project mainly focus on maintaining animal welfare during the process of obtaining the faecal sample per rectum. Methods for obtaining the faecal samples have been refined to minimise any potential stress or discomfort. The samples are only obtained after the cats are anaesthetised for the sterilisation procedure, during the scrubbing process where urine and faeces are commonly expressed so that the operating field is not contaminated. Prior to scrubbing, premedication drugs which include sedatives and analgesics are also administered in preparation for the procedure. Hence, the cats were unconscious during the sample collection and would not feel any pain. Moreover, taking rectal temperatures is a standard procedure done during a physical examination, and digital rectal examinations are appropriate in the case of suggestive clinical signs in cats.^15^ These procedures do not harm the cat, and it has been shown that there is minimal distress during these procedures.^15^ Other measures taken to minimise potential stress included using a Feliway‐sprayed room to keep the cats calm during their stay at TAHMU. While there are no methods to replace or partially replace the use of cats, the study aims to use the smallest number of cats possible while still obtaining satisfactory levels of information. The study was approved by Murdoch University Animal Ethics committee, Protocol ID 1058, Permit Number R3501/23.

The laboratory protocol is summarised in Figure 1. Faecal samples were streaked, using the streak plate technique, onto various *E. coli*‐specific agar, including CHROMagar ECC plates, CHROMagar ECC + ciprofloxacin plates, CHROMagar ECC + ampicillin plates, as well as extended‐spectrum beta‐lactamases (ESBL) growth media plates. A sterile disposable inoculating loop was used to spread a small volume of a bacterial sample across the surface of an agar plate in a sequential pattern (typically in quadrants), with sterilisation of the loop between streaking steps. This process progressively diluted the bacterial inoculum across the plate, allowing individual bacterial cells to become spatially separated. Following incubation at 37°C for 24 h, discrete colonies arising from single cells or colony‐forming units were selected to represent pure cultures for further analysis.

**Figure 1:**
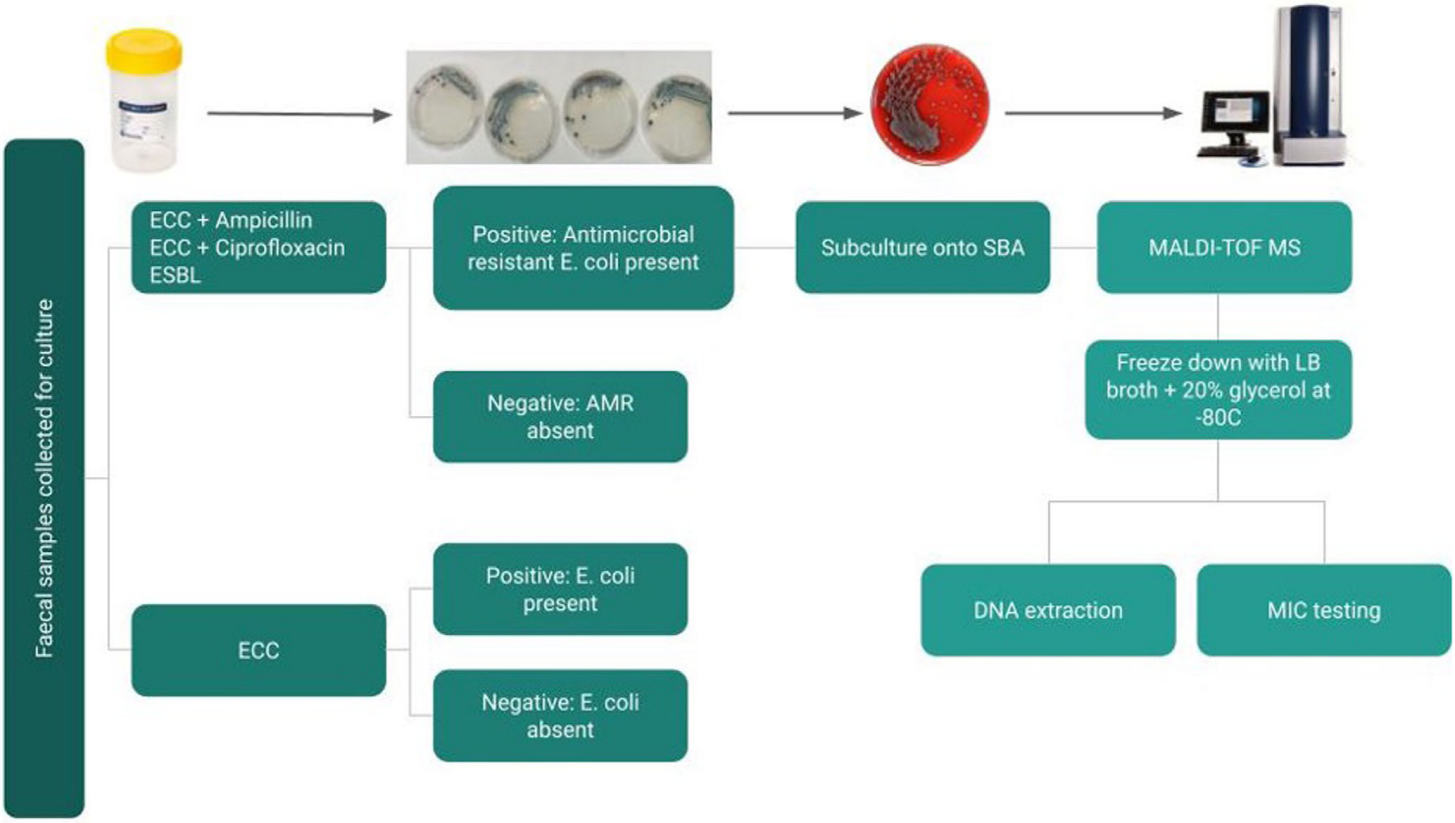
Flowchart of the laboratory protocol used. ECC, *E. coli* and other coliforms; ESBL, extended‐spectrum beta‐lactamases; SBA, sheep blood agar; MALDI‐TOF MS, Matrix Assisted Laser Desorption Ionization‐Time Of Flight Mass Spectrometry; LB, Luria–Bertani; MIC, minimum inhibitory concentration.

Colony growth on the CHROMagar ECC plates indicated the presence of *E. coli* in the faecal samples and hence in the gut of the cats. On the other hand, colony growth in the antimicrobial‐supplemented plates (CHROMagar ECC + ampicillin plates, CHROMagar ECC + ciprofloxacin plates) indicated the presence of *E. coli* resistant to the respective antimicrobial. Colony growth on the ESBL plates suggests that *E. coli* isolated produced ESBL enzymes. Growth characteristics of positive cultures include blue and/or pink colonies in CHROMagar ECC and the antimicrobial‐infused plates, and yellow colonies in ESBL plates. A single colony from the positive cultures with AMR was then sub‐isolated onto sheep blood agar (SBA) and incubated at 37°C for 24 h. Sub‐isolation was done with the same 16 streak technique, using disposable sterile inoculation loops.

The sub‐isolated colonies were subsequently analysed via Matrix Assisted Laser Desorption Ionization‐Time Of Flight Mass Spectrometry (MALDI‐TOF MS) testing. This identified the species of bacteria present and confirmed the presence of antimicrobial‐resistant *E. coli*. The antimicrobial‐resistant isolates were frozen in 2 mL sterile plastic screw‐cap vials containing Luria–Bertani (LB) broth and 20% glycerol and were stored at −80°C for future minimum inhibitory concentration (MIC) testing and potential DNA extraction for sequencing of resistance genes. LB broth contains nutrients for *E. coli* cultivation^16^ and is suitable for the maintenance and storage of *E. coli*.^17^ The presence of glycerol in the freeze down medium acts as a cryoprotectant^18^ and helps to stabilise frozen bacteria by protecting the cell membranes from damage,^19^ ensuring that the bacteria remain viable. The stock of bacteria can be reliably stored at −80°C for up to 10 years.^20^

MIC testing was also conducted to assess the clinical breakpoints and antimicrobial susceptibility of the *E. coli*. The stock of antimicrobial‐resistant *E. coli* frozen in LB broth at −80°C was inoculated onto SBA with the streak plate technique, using disposable sterile inoculation loops. The agar plates were incubated for 24 h at 37°C to allow for bacterial growth and then tested against a panel of antimicrobials according to the CLSI guidelines. The antimicrobial‐resistant *E. coli* isolates were tested against a panel of 13 antimicrobial agents using broth microdilution. The concentration ranges tested (μg/mL) were as follows: amikacin (1–64), ampicillin (1–32), cefotaxime (0.015–4), ceftazidime (0.125–16), chloramphenicol (2–32), ciprofloxacin (0.008–2), colistin (0.25–8), florfenicol (4–32), gentamicin (0.25–16), meropenem (0.008–4), sulfamethoxazole (8–512), tetracycline (1–32), and trimethoprim (0.25–16). These agents were selected to encompass a spectrum ranging from first line to critically important antimicrobials (CIAs), thereby enabling a comprehensive assessment of the resistance profiles of the *E. coli* isolates.

## Results

A total of 72 faecal samples were cultured on CHROMagar ECC plates, yielding *E. coli* growth from 69 samples (95.8%, 69/72). Of these, 26 samples (37.7%, 26/69) produced growth on CHROMagar ECC + ampicillin plates, indicating the presence of ampicillin‐resistant *E. coli*. One sample (1.4%, 1/69) produced growth on ESBL plates, indicating the presence of an ESBL‐producing *E. coli*. No growth was detected on CHROMagar ECC + ciprofloxacin plates (0%, 0/69), indicating an absence of ciprofloxacin‐resistant *E. coli* in the sampled population.

A total of 27 isolates were selected for MIC testing: one isolate per ampicillin‐resistant positive sample (*n* = 26) and the single ESBL‐producing isolate (*n* = 1). The percentage distribution of antimicrobial susceptibility profiles from MIC testing is shown in Figure 2 and the categorical susceptibility patterns are presented in Table 1. Ampicillin resistance was detected among all 27 isolates (100%, 27/27). Resistance to tetracycline (37.0%, 10/27), sulfamethoxazole (33.3%, 9/27), and trimethoprim (29.6%, 8/27) was also detected. Low resistance rates to third‐generation cephalosporins were observed, with 7.4% (2/27) of isolates resistant to both cefotaxime and ceftazidime respectively. One isolate (3.7%, 1/27) demonstrated an ESBL phenotype. No resistance was detected to ciprofloxacin, colistin, meropenem, chloramphenicol, florfenicol, gentamicin, or amikacin (0%, 0/27).

**Figure 2:**
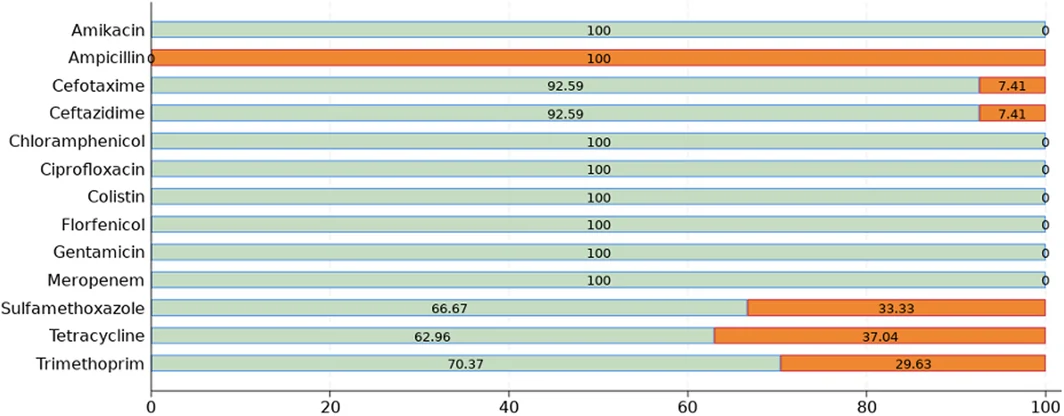
Percentage distribution of antimicrobial susceptibility profiles among 27 bacterial isolates tested against 13 antibiotics. Stacked horizontal bars represent the proportion of isolates classified as susceptible (green) and resistant (orange) to each antimicrobial agent.

**Table 1:** Distribution of minimum inhibitory concentrations (MICs) and categorical susceptibility results for 27 bacterial isolates tested against 13 antimicrobial agents.

Antimicrobial	*n*	0.008	0.016	0.031	0.063	0.13	0.25	0.5	1	2	4
Amikacin	27								77.78	22.22	
Ampicillin	27										
Cefotaxime	27				66.67	3.7		11.11	7.41	3.7	
Ceftazidime	27						37.04	33.33	22.22		
Chloramphenicol	27										
Ciprofloxacin	27		100								
Colistin	27						100				
Florfenicol	27										25.93
Gentamicin	27						74.07	22.22	3.7		
Meropenem	27		3.7	85.19	11.11						
Sulfamethoxazole	27										
Tetracycline	27									59.26	3.7
Trimethoprim	27						11.11	40.74	14.81	3.7	

Antimicrobial	8	16	32	64	128	256	512	1024	2048	*R* (%)	ci
Amikacin										0.00	0–12.77
Ampicillin				100						100.00	87.23–100
Cefotaxime	7.41									7.41	0.91–24.29
Ceftazidime		7.41								7.41	0.91–24.29
Chloramphenicol										0.00	0–12.77
Ciprofloxacin										0.00	0–12.77
Colistin										0.00	0–12.77
Florfenicol	74.07									0.00	0–12.77
Gentamicin										0.00	0–12.77
Meropenem										0.00	0–12.77
Sulfamethoxazole	11.11	18.52	18.52	18.52				33.33		33.33	16.52–53.96
Tetracycline			3.7	33.33						37.04	19.4–57.63
Trimethoprim			29.63							29.63	13.75–50.18

Horizontal bars represent the percentage of isolates at each MIC concentration (μg/mL), with vertical lines indicating established clinical breakpoints. Shaded areas in the table indicate the concentration ranges tested for each antimicrobial. The right panel summarises the proportion of isolates classified as resistant (R), along with corresponding 95% confidence intervals.

Antimicrobial resistance profiles based on antimicrobial classes of *E. coli* isolates subjected to MIC testing is shown in Table 2. Of the 27 isolates, 14 (51.9%) carried resistance to beta‐lactams alone (bla). Two isolates (7.4%) each commonly detected co‐resistance to beta‐lactams and third‐generation cephalosporins (bla_c3g) and beta‐lactams and tetracyclines (bla_tet), respectively. One isolate (3.7%) demonstrated co‐resistance to beta‐lactams and folate pathway inhibitors (bla_fpi). Resistance to three or more antimicrobial classes was observed in eight isolates (29.6%, 8/27), reflecting co‐resistance to beta‐lactams, folate pathway inhibitors, and tetracyclines (bla_fpi_tet).

**Table 2:** Antimicrobial resistance profiles of *E. coli* isolates by antimicrobial class.

Phenotype	*n*	Frequency (%)
1: Bla	14	51.8519
2: bla_c3g	2	7.4074
2: bla_fpi	1	3.7037
2: bla_tet	2	7.4074
3: bla_fpi_tet	8	29.6296

bla, beta‐lactam resistance; c3g, third‐generation cephalosporin resistance; fpi, folate pathway inhibitor resistance; tet, tetracycline resistance.

## Discussion

The present study investigated the carriage of antimicrobial‐resistant *E. coli* among urban stray cats in Perth, Western Australia, demonstrating that resistant *E. coli* is detectable in this population despite their presumed low direct antimicrobial exposure. Notably, no ciprofloxacin or fluoroquinolone resistance was detected and only low‐level resistance to extended‐spectrum cephalosporins was observed, suggesting that the immediate CIA‐related public health risk within this population appears limited at this time. However, ampicillin resistance was common, occurring in 37.7% (26/69) of samples yielding *E. coli*, indicating that first‐line antimicrobial resistance is well established within this population. These findings should be interpreted with caution, as the cohort was drawn exclusively from a single shelter admission program and extrapolation to the broader urban stray cat population requires further epidemiological investigation.

Ampicillin exhibited the highest prevalence of phenotypic AMR in stray cats. Ampicillin is an antimicrobial within the class of penicillin,^21^ which are broad spectrum antimicrobials, often used as the first‐line antimicrobial in Australia in the form of potentiated aminopenicillins^22^ for small animals. An initial low‐level resistance frequently serves as a precursor to the emergence of high‐level resistance.^23^ With such a capacity of *E. coli* to survive in the presence of high concentrations of ampicillin, this suggests considerable AMR and thus results in restricted choices in drugs to treat pathologies caused by the resistant pathogen.^24^ As ampicillin is a first‐line antimicrobial used in veterinary medicine, the prevalence of AMR increases the risk of penicillins being less reliable and effective in treating bacterial infections in animals. Ultimately, if the resistant population of *E. coli* supplants the susceptible wild bacterial population in the environment, it achieves long‐term stability within its ecosystem^25^ and becomes a major threat to the health of animals and humans.

Previous studies have reported that urban pet cats in Australia roam approximately 0.01–0.64 ha per day, while rural pet cats range from 0.07–2.86 ha.^26^ In comparison, feral cats in rural Western Australia have been recorded to roam between 9 and 34 ha per day.^12^ However, published data describing the roaming behaviour of urban stray cats in Perth are currently lacking. While pet cats generally occupy smaller daily ranges, urban stray cats may potentially traverse larger or variable areas, increasing the likelihood of spatial overlap with owned cats. Such overlap could provide opportunities for indirect or direct contact between stray and domestic cats, thereby facilitating the potential transmission of antimicrobial‐resistant *E. coli*. On this basis, urban stray cats may represent a possible reservoir of ampicillin‐resistant *E. coli*. However, further epidemiological and transmission studies are required to substantiate this potential role and to quantify the associated risk to domestic animal populations. At present, there is limited published evidence characterising the frequency and nature of interactions between urban stray cats, domestic animals, and humans, which may represent key pathways for the transmission of AMR *E. coli*. Future studies investigating the prevalence and context of stray–domestic animal and stray–human contact would assist in more accurately determining the risk and directionality of AMR *E. coli* transmission across these populations. A similar study in cats in Italy found the most common AMR in *E. coli* was to ampicillin.^11^ This supports the likelihood that ampicillin‐resistant *E. coli* could be a global issue. In light of the findings, it is recommended that more studies are done in other regions of the world to evaluate if this is a common occurrence and whether there are any similar trends.

Another study conducted in Australia reported that Australian silver gulls (*Chroicocephalus novaehollandiae*) carried *E. coli* isolates resistant to ampicillin and other CIAs, including ciprofloxacin and ceftriaxone.^27^ These findings suggest that silver gulls may act as environmental reservoirs and dispersal agents of CIA‐resistant bacteria across urban and coastal environments. As urban stray cats frequently occupy similar anthropogenic habitats, including urban areas, waste sites, and coastal regions where gulls forage, overlap in these environments may facilitate indirect exposure of cats to antimicrobial‐resistant *E. coli*. Consequently, environmental acquisition from wildlife reservoirs such as silver gulls may contribute to the presence of resistant bacteria in urban stray cats, which may themselves act as reservoirs for antimicrobial‐resistant *E. coli* within urban ecosystems.

Only one ESBL producing *E. coli* was recovered (1.4%, 1/69). When *E. coli* produces ESBL enzymes, they can hydrolyze and cause cleavage of the beta‐lactam ring system,^28^ conferring resistance to antimicrobials such as penicillins and cephalosporins.^29^ With a low percentage of ESBL‐producing *E. coli*, beta‐lactams (with the exception of ampicillin and potentially penicillins) can still be considered a reliable antimicrobial in treating *E. coli* infections in urban stray cats. However, ESBL genes are plasmid‐mediated and can be easily transmitted among Enterobacteriaceae species.^30^ ESBL genes in animals can also be transferred horizontally to pathogenic strains and potentially undergo further zoonotic spread to humans through faecal‐oral contamination.^31^, ^32^, ^33^ The shedding of ESBL producing *E. coli* in faeces can therefore contribute to their spread in households, veterinary clinics, and the environment. With the ease of spread of resistance, treating infections becomes more challenging, and the reservoir of beta‐lactam‐resistant *E. coli* may continue to expand in the population of urban stray cats even without them showing signs of infection. Continuous monitoring for ESBL prevalence in healthy cats will be useful for tracking antimicrobial resistance trends and can inform future infection control strategies.

No ciprofloxacin resistance carriage was detected among the *E. coli* from cats in this study. Ciprofloxacin, a fluoroquinolone antimicrobial, is generally reserved as a second‐line agent in veterinary medicine and is often prescribed following bacterial culture and susceptibility testing.^34^ Its comparatively restricted use may minimise selective pressure for the development and maintenance of ciprofloxacin resistance in bacterial populations.^35^

The cats recruited for this study were clinically healthy young stray animals for which no prior medical history was available. Although their previous antimicrobial exposure could not be determined, the absence of detected ciprofloxacin resistance in this cohort may be consistent with limited fluoroquinolone exposure within this population. Nevertheless, antimicrobial resistance to last‐line agents has been documented in feline populations. A previous study reported the detection of carbapenem‐resistant *Salmonella enterica* serotype Typhimurium in a clinically ill cat and three additional cats within a shelter environment.^36^ This demonstrates that companion animals can harbor resistance to CIAs, potentially independent of documented direct antimicrobial administration. *Salmonella* spp. and *E. coli* are both Gram‐negative, facultative anaerobic bacteria within the family Enterobacteriaceae and share several resistance mechanisms.^37^ Accordingly, the emergence of resistance to CIAs, such as carbapenems, in Salmonella highlights the broader capacity for Enterobacteriaceae to acquire and disseminate such resistance determinants. Dogs and cats in Australia also have been identified as potential spillover hosts of ciprofloxacin‐resistant *E. coli*, suggesting possible interspecies transmission dynamics involving companion animals.^38^ Although no ciprofloxacin resistance was identified in *E. coli* isolates in the present study, the potential for acquisition of resistance to CIAs cannot be excluded

Geographic variation in resistance patterns should also be considered. Although no ciprofloxacin‐resistant *E. coli* isolates were identified in this study, fluoroquinolone resistance in *E. coli* from cats has been reported in diverse geographic regions. A study conducted in Thailand reported fluoroquinolone resistance among *E. coli* isolates from pet cats and their drinking water.^39^ Another study conducted in Japan investigating sheltered dogs and cats reported that 17.9% (14/78) of feline faecal samples yielded fluoroquinolone‐resistant *E. coli* isolates,^40^ underscoring regional differences in antimicrobial resistance profiles and the broader complexity of AMR epidemiology in feline populations. Furthermore, a study in Australia demonstrated that CIA‐resistant *E. coli* in Australian silver gulls (*Chroicocephalus novaehollandiae*) were detected more frequently in urban areas compared to rural settings,^41^ supporting an association between anthropogenic environmental exposure and the acquisition of resistant bacteria in wildlife. These findings may have relevance to stray cats residing in urban environments, where increased exposure to human‐associated settings and urban wildlife could potentially contribute to the acquisition and dissemination of CIA‐resistant *E. coli*. However, direct transmission pathways remain to be elucidated.

Lastly, the detection of clinical resistance to sulfamethoxazole (33.33%), tetracycline (37.04%), and trimethoprim (29.63%) in ampicillin‐resistant *E. coli* isolates underscores a significant concern in both veterinary medicine and public health. This suggests a high prevalence of multi‐drug resistance (MDR) in *E. coli*. This MDR pattern is particularly pertinent in the context of urban stray cats, which often serve as reservoirs for antimicrobial‐resistant bacteria due to their exposure to diverse environmental sources and lack of medical oversight.^11^, ^42^ These include contact with contaminated water sources,^43^ and possibly the ingestion of improperly disposed human or veterinary medications, as well as consumption of food waste containing antibiotic residues. Additionally, interactions with other animals carrying resistant bacteria can facilitate the transfer of resistance genes. Such environmental exposures will contribute to the development and spread of MDR bacteria among stray cat populations. The admission of urban stray cats into veterinary settings can introduce these resistant strains into the clinical environment, posing risks to other animals and potentially to humans. The close contact between veterinary staff and animals facilitates the transmission of resistant bacteria, emphasizing the need for stringent hygiene and biosecurity measures. Moreover, the presence of MDR *E. coli* complicates treatment options, as commonly used antibiotics may be ineffective, leading to prolonged infections and increased costs. Addressing this issue requires a multifaceted approach. Implementing routine antimicrobial susceptibility testing to guide effective treatment, enforcing strict infection control protocols within veterinary practices, and monitoring antimicrobial resistance patterns in stray animal populations would be crucial to mitigate the spread of MDR bacteria and safeguard both animal and public health.

This study has several limitations. The cross‐sectional design of this study limited the ability to assess temporal trends or seasonal variation in antimicrobial‐resistant (AMR) *Escherichia coli* carriage among urban stray cats. Seasonal differences in bacterial growth and horizontal gene transfer have been associated with increased temperatures,^44^ which may influence AMR dynamics. However, sample numbers varied between months due to fluctuations in the number of cats admitted to the spay–neuter programme. Although at least one sample was collected each month, smaller sample sizes during certain periods may have limited the ability to accurately assess seasonal patterns.

Another limitation was the absence of medical histories for the urban stray cats, preventing determination of prior antimicrobial exposure. Although the cats were juveniles (≤1 year old) typically admitted as clinically healthy stray kittens, previous antimicrobial treatment cannot be excluded. Any prior exposure may have exerted selective pressure contributing to the emergence of AMR.^45^

Several methodological considerations should also be acknowledged. Cats were sampled through a large shelter with a broad geographic catchment, which provided a substantial and diverse cohort but may not fully represent the wider stray population. Additionally, the absence of molecular confirmation of ESBL genes limited the ability to characterise the genetic determinants underlying the observed phenotypic resistance patterns. Finally, analysis was based on a single *E. coli* isolate per animal, which may underestimate within‐host strain diversity and the potential coexistence of multiple resistance phenotypes.

Future studies could incorporate genomic characterisation of resistant isolates to identify resistance genes and determine whether resistance arises through horizontal gene transfer or chromosomal mutations.^46^ Identification of plasmid‐mediated resistance genes would be particularly important due to their potential for rapid dissemination.^47^ Expanding the antimicrobial panel and investigating interactions between stray and owned cats could further clarify the role of urban stray cats as reservoirs and potential sources of AMR transmission.

Although urban stray cats are presumed to experience relatively low direct antimicrobial selection pressure, the detection of AMR *E. coli* within this population suggests that resistance may be acquired through environmental exposure in urban ecosystems. The notable prevalence of resistance to commonly used antimicrobials, particularly ampicillin, highlights the importance of a One Health approach to addressing antimicrobial resistance. Continued surveillance and monitoring of phenotypic and genotypic resistance across human, animal, and environmental interfaces will be essential to preserve the effectiveness of antimicrobial agents in both veterinary and human medicine.^48^, ^49^, ^50^

## Conflicts of interest and sources of funding

The authors declare no conflict of interest. This research was funded by The School of Veterinary Medicine, Murdoch University, and the AMRID Laboratory.

## Data Availability

The data that support the findings of this study are available from the corresponding author upon reasonable request.
